# Supplementary material for: Optimised Agrobacterium-Mediated Transformation and Application of Developmental Regulators Improve Regeneration Efficiency in Melons
Source: Genes (Basel). 2023 Jul 12;14(7):1432. doi: 10.3390/genes14071432 (PMC10378916; doi:10.3390/genes14071432)
Supplement: Supplementary file 1 [file genes-14-01432-s001.zip › genes-2431510-supplementary.pdf]

Supplementary Information

Optimised *Agrobacterium*-mediated Transformation and Application of Developmental Regulators Improve Regeneration Efficiency in Melons

Lili Wan [1+], Zhuanrong Wang [1+], Xuejun Zhang [2,3], Hongxia Zeng [1], Jian Ren [1], Na Zhang [1], Yuhong Sun [1] and Tang Mi [1*]

cgacactctcgtctactccaagaatatcaaagatacagtctcagaagaccaaagggctattgagacttttcaacaaagggtaatatcgggaaacctcctcggattccatt
gcccagctatctgtcacttcatcaaaaggacagtagaaaaggaaggtggcacctacaaatgccatcattgcgataaaggaaaggctatcgttcaagatgcctctgccg
acagtggtcccaaagatggacccccacccacgaggagcatcgtggaaaaagaagacgttccaaccacgtcttcaaagcaagtggattgatgtgaacatggtggagc
acgacactctcgtctactccaagaatatcaaagatacagtctcagaagaccaaagggctattgagacttttcaacaaagggtaatatcgggaaacctcctcggattccat
tgcccagctatctgtcacttcatcaaaaggacagtagaaaaggaaggtggcacctacaaatgccatcattgcgataaaggaaaggctatcgttcaagatgcctctgccg
acagtggtcccaaagatggacccccacccacgaggagcatcgtggaaaaagaagacgttccaaccacgtcttcaaagcaagtggattgatgtgatatctccactgac
gtaagggatgacgcacaatcccactatccttcgcaagacccttcctctatataaggaagttcatttcatttggagaggacacgctgaaatcaccagtctctctctacaaatc
tatctctcaaacattactcattcacaaaaccatcttaaagcaactacacaagtcttgaaattttctcatattttctatttactatataaacttttaatcaaatcaagattaactATG
ACAACCTTCAAAATCGAGTCCCGGATCCACGGCAACCTCAACGGGGAGAAGTTCGAGTTGGTTGGAG
GTGGAGTAGGTGAGGAGGGTCGCCTCGAGATTGAGATGAAGACTAAAGATAAACCACTGGCATTCTC
TCCCTTCCTGCTGACCACTTGCATGGGTTACGGGTTCTACCACTTCGCCAGCTTCCCAAAGGGGATTA
AGAACATCTATCTTCATGCTGCAACGAACGGAGGTTACACCAACACCAGGAAGGAGATCTATGAAGA
CGGCGGCATCTTGGAGGTCAACTTCCGTTACACTTACGAGTTCAACAAGATCATCGGTGACGTCGAGT
GCATTGGACATGGATTCCCAAGTCAGAGTCCGATCTTCAAGGACACGATCGTGAAGAGTTGTCCCACG
GTGGACCTGATGTTGCCAATGTCCGGGAACATCATCGCCAGCTCCTACGCTTACGCCTTCCAACTGAA
GGACGGCTCTTTCTACACGGCAGAAGTCAAGAACAACATAGACTTCAAGAATCCAATCCACGAGTCC
TTCTCGAAGTCAGGGCCCATGTTCACCCACAGACGTGTCGAGGAGACTCTCACCAAGGAGAACCTTG
CCATAGTGGAGTACCAGCAGGTTTTCAACAGCGCCCCAAGAGACATGTAGatatgaagatgaagatgaaatatttggtg
tgtcaaataaaaagcttgtgtgcttaagtttgtgttttttcttggcttgttgtgttatgaatttgtggctttttctaatattaaatgaatgtaagatctcattataatgaataaacaaat
gtttctataatccattgtgaatgttttgttggatctcttctgcagcatataactactgtatgtgctatggtatggactatggaatatgattaaagataagatgggctcatagagta
aaacgaggcgagggacctataaacctcccttcatcatgctatttcatgatctattttataaaataaagatgtagaaaaaagtaagcgtaataaccgcaaaacaaatgattta
aaacatggcacataatgaggagattaagttcggtttacgtttattttagtactaattgtaacgtgagactacgtatcgggaatcgcctaattaaagcattaatgcgaacctga
ttagattcaccgaccctcctatcgtgtcgacctttctgtttcttagaatttttggtagtctatgtactaataatgtcagcttcgtatttatttcataagcaatttgcatttgcaatttgt
tttttacttttatttttattgtattgtggaatgtggactcgtaccaacatgaagttatataccaccaaaaaaattacagttagtcaaaagattcacgagtgagagctacttatgatt
gtcttttacgtatatgtctaattgtctatttgctcaataatctttgtactttcttttgtcgttgataaaatcacaaagttccaaaagtaatcgaatgatttgcttttaagaaaagaaga
gctcaataattcaacatatatctgtacaca

Supplemental Figure S1. CpYGFP_eYGFPuv expression cassette in the binary vector PV16
Black indicates the CaMV35S promoter, blue indicates the 5′-UTR, red indicates CpYGFP_eYGFPuv, green indicates the 3′-UTR of At5g59720, purple indicates the At5g59720 terminator.

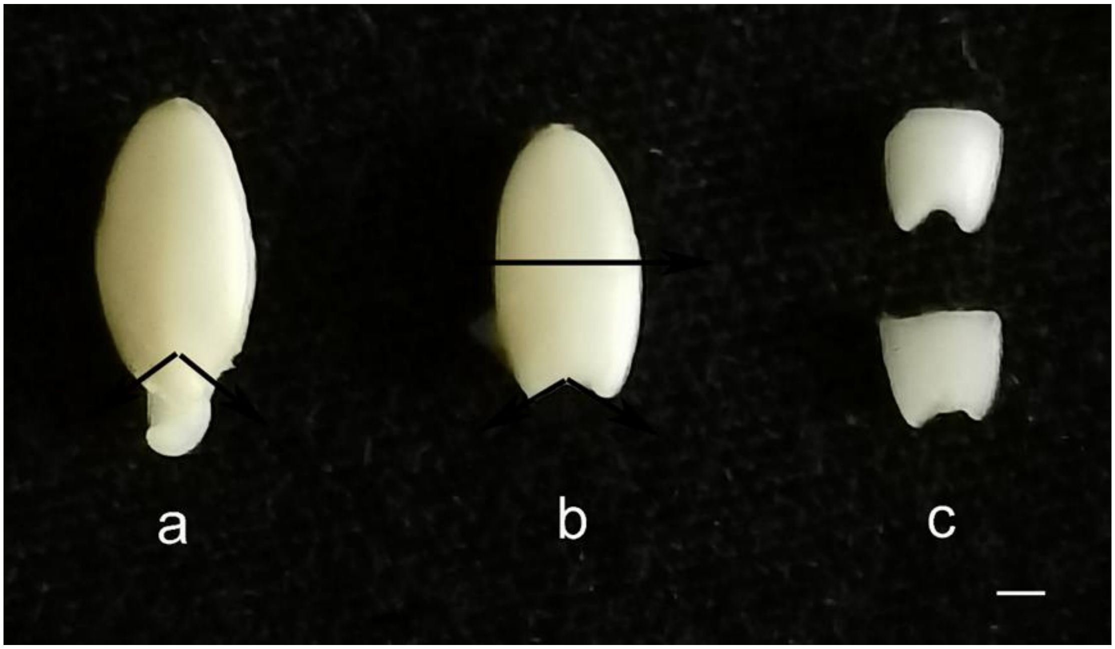

Supplemental Figure S2. Explant preparation
Excision of embryos from the germinated seeds (a). Cotyledons were cut in half transversely (b) and proximal parts with U-shaped ends were selected as explants (c). Bar = 0.5 cm

Table S1. Primers used in this study

Primer name	Sequence (5′–3′)	
PAtGRF5-F	TTACATTTACAATTACCATGGATGATGAGTCTAAGTGGAA	
PAtGRF5-R	CTCTAGACTCACCTAGGATCCTTAGCTACCAGTGTCGAGT	
PAtPLT5-F	TTACATTTACAATTACCATGGATGAAGAACAATAACAACA	
PAtPLT5-R	CTCTAGACTCACCTAGGATCCTCATTCCAACCCAAAAACC	
PAtBBM-F	TTACATTTACAATTACCATGG ATGAACTCGATGAATAACTG	
PAtBBM-R	CTCTAGACTCACCTAGGATCCCTAAGTGTCGTTCCAAACTG	
PAtWUS-F	TTACATTTACAATTACCATGGATGGAGCCGCCACAGCATC	
PAtWUS-R	CTCTAGACTCACCTAGGATCCCTAGTTCAGACGTAGCTCA	
PAtWOX5-F	TTACATTTACAATTACCATGGATGTCTTTCTCCGTGAAAGG	
PAtWOX5-R	CTCTAGACTCACCTAGGATCCTTAAAGAAAGCTTAATCGA	
PAtWIND1-F	TTACATTTACAATTACCATGGATGGCAGCTGCTATGAATT	
PAtWIND1-R	CTCTAGACTCACCTAGGATCCCTAAGCTAGAATCGAATCC	
Primers for the construction of gRNA expression vectors targeting CmPDS		
T1CmPDS-319F:	GTATCCGGTCTCGATTGATAGTGAGATTGTGGGCGATGTT	
T1CmPDS-319F0:	TGATAGTGAGATTGTGGGCGATGTTTTAGAGCTAGAAATAGC	
T2CmPDS-320R0:	AACTAGACCACAGATAGATGATACAATCACTACTTCGTCTCTAA	
T2CmPDS-320R:	TAGATTGGTCTCGAAACTAGACCACAGATAGATGATACAA	
Primers for Hi-Tom		
CmPDS-F373	GGAGTGAGTACGGTGTGCGTTCTCCATTAAGTTGTGAG	first-round PCR
CmPDS-R373	GAGTTGGATGCTGGATGGACTAGCAGATAAGGAAGCTG	
HT-In-20F	AATGATACGGCGACCACCGAGATCTACACCTTGACAGACACTC TTTCCCTACACGACGCTCTTCCG	second-round PCR
HT-In-20R	CAAGCAGAAGACGGCATACGAGATACGTGATAGTGACTGGAGT TCAGACGTGTGCTCTT	
Primers for genotyping the transformed seedlings		
Bar-354F	GATTAGGCATCGAACCTTCA	
Bar-354R	CTATCCCTCGCTTCCTTCTC	
PB7-AtU6-26F	GATTAGGCATCGAACCTTCA	
PB7-AtU6-26F	CTATCCCTCGCTTCCTTCTC	

Table S2 Mutation types determined by Hi-TOM

A01 #ZHF line1 Target 1 (Homozygous mutation)

Sort	Read number	Ratio	Left variation type	Left variation	Right variation type	Right variation
1	2315	95%	3D	TGG	3D	TGG

C02 #ZHF line 11 Target 1 (heterozygous mutation)

Sort	Read number	Ratio	Left variation type	Left variation	Right variation type	Right variation
1	1125	52%	6D	TGGGCG	6D	TGGGCG
2	1057	48%	-	WT	-	WT

D5 #Z12 line 3 Target 1 (Homozygous mutation)

Sort	Read number	Ratio	Left variation type	Left variation	Right variation type	Right variation
1	1891	96%	5D	GTGGG	5D	GTGGG

F2 #Z12 line 9 Target 1 (Heterozygous mutation)

Sort	Read number	Ratio	Left variation type	Left variation	Right variation type	Right variation
1	1563	54%	6D	GTGGGC	6D	GTGGGC
2	1338	46%	-	WT	-	WT

B05 #ZHF line4 Target 2 (Heterozygous mutation)

Sort	Read number	Ratio	Left variation type	Left variation	Right variation type	Right variation
1	1455	52%	1D 2I	A >GC	1D 2I	A >GC
2	1326	48%	3D	CAC	3D	CAC

C06 #Z12 line10 Target 2 (Heterozygous mutation)

Sort	Read number	Ratio	Left variation type	Left variation	Right variation type	Right variation
1	1231	55%	1D	A	1D	A
2	1018	45%	3D	CAC	3D	CAC

Table S3 Survival rate of the melon variety ZHF after Treatments A, B, C, and D

Treatment A	ZHF		
	No. of surviving explants	No. of explants	survival rate %
Rep 1	81	90	90
Rep 2	83	87	95
Rep 3	76	89	85
Average of survival rate %			90±5a
Treatment B	ZHF		
	No. of surviving explants	No. of explants	survival rate %
Rep 1	94	110	85
Rep 2	85	109	78
Rep 3	91	101	90
Average survival rate %			84±6a
Treatment C	ZHF		
	No. of surviving explants	No. of explants	survival rate %
Rep 1	94	110	85
Rep 2	80	109	73
Rep 3	68	98	69
Average survival rate %			75±8a
Treatment D	ZHF		
	No. of surviving explants	No. of explants	survival rate %
Rep 1	76	110	69
Rep 2	63	102	62
Rep 3	74	105	70
Average survival rate %			67±4b

The number of explants is the sum of surviving explants after infection in three independent experiments. The average survival rate (%) represents the mean of three independent replicates. Different lowercase letters indicate significant differences ($P < 0.1$, Tukey's test). Treatment A: micro-brushing (Brush) + sonication (Son) (30 s); treatment B: Brush + Son (30 s) + vacuum (-0.3 kPa); treatment C: Brush + Son (30 s) + vacuum (-0.5 kPa); treatment D: Brush + Son (30 s) + vacuum (-1.0 kPa).

Table S4 Survival rate of melon variety Z12 after Treatments A, B, C, and D

Treatment A	Z12		
	No. of surviving explants	No. of explants	survival rate %
Rep 1	84	88	95
Rep 2	95	104	91
Rep 3	90	98	92
Average survival rate %			93±3a
Treatment B	Z12		
	No. of surviving explants	No. of explants	survival rate %
Rep 1	78	87	90
Rep 2	81	92	88
Rep 3	80	95	84
Average survival rate %			87±3a
Treatment C	Z12		
	No. of surviving explants	No. of explants	survival rate %
Rep 1	75	99	76
Rep 2	78	101	77
Rep 3	84	112	75
Average survival rate %			76±1b
Treatment D	Z12		
	No. of surviving explants	No. of explants	survival rate %
Rep 1	71	109	65
Rep 2	71	102	70
Rep 3	62	103	60
Average survival rate %			65±5c

The number of explants is the sum of the surviving explants after infection in three independent experiments. The average survival rate (%) represents the mean of three independent replicates. Different lowercase letters indicate significant differences ($P < 0.1$, Tukey's test). Treatment A: micro-brushing (Brush) + sonication (Son) (30 s); treatment B: Brush + Son (30 s) + vacuum (-0.3 kPa); treatment C: Brush + Son (30 s) + vacuum (-0.5 kPa); treatment D: Brush + Son (30 s) + vacuum (-1.0 kPa).

Supplemental File S1. Tissue culture medium
Germination medium: 4.4 g/L Murashige and Skoog (MS) medium (PhytoTech LABS, M519), 30 g/L sucrose, 0.5 g/L Phytagel (Sigma-Aldrich, P8169), pH 5.85.
Inoculation medium: 1 mg/L 6-benzylaminopurine (6BA), 1 mg/L abscisic acid (ABA), 200 μM acetosyringone, and 1.25 mM morpholinoethanesulfonic acid were added to MS medium, 30 g/L sucrose, pH 5.40.
Co-cultivation medium: 0.5 mg/L 6BA, 1 mg/L ABA, 200 μM AS, and 1.25 mM morpholinoethanesulfonic acid were added to MS medium, 30 g/L sucrose, pH 5.40.
Selection medium: 1 mg/L 6BA, 0.1 mg/L ABA, 1 mg/L $CuSO4·5H_2O$, 150 mg/L timentin, 250 mg/L cefotaxime, and 4 mg/L glufosinate-ammonium were added to MS medium, 30 g/L sucrose, pH 5.8.
Root induction medium:1 mg/L indole-3-butyric acid, 150 mg/L timentin, and 250 mg/L cefotaxime were added to MS medium, 30 g/L sucrose, pH 5.8.
To prepare the liquid medium for *Agrobacterium* culture, the LB medium was supplemented with spectinomycin (50 mg/L) and rifampicin (25 mg/L). All tissue cultures were maintained in a culture room under a 16-h light/8-h dark cycle at 22°C to 25°C.
